# Spontaneous bilateral quadriceps tendon rupture: a case report

**DOI:** 10.11604/pamj.2020.37.84.22329

**Published:** 2020-09-23

**Authors:** Kelechukwu Mc’Clement Onuoha, Olubukola Khadija Ajiboye, Rajeev Kumar

**Affiliations:** 1 Cedarcrest Hospitals Abuja, Abuja, Nigeria

**Keywords:** Quadriceps tendon rupture, proximal myopathy, transosseous

## Abstract

Quadriceps tendon rupture is an uncommon injury and mostly occurs among middle aged individuals that are involved in sports such as running or jumping. Spontaneous bilateral quadriceps tendon rupture is a rarer injury, however, can be debilitating. Patients with such injuries usually present with history of minor trauma, swelling and inability to actively extend the knee. Its occurrence secondary to minor trauma is mostly associated with chronic diseases and long-term use of certain medications. Occurrence of simultaneous bilateral quadriceps tendon rupture in the absence of trauma in a healthy patient with no known medical condition is yet to be reported and therefore requires a high index of suspicion for early diagnosis and effective management to avoid complications. The aim of this case report is to create awareness of the spontaneous occurrence of this injury in the absence of the reported risk factors. We report the unusual case of a 60-year old healthy man who presented with a spontaneous simultaneous bilateral quadriceps' tendon rupture in the absence of trauma and no medical risk factors. We report the unusual case of a 60-year old healthy man who presented with a spontaneous simultaneous bilateral quadriceps tendon rupture in the absence of trauma and no medical risk factors. Consent was taken from him to be used as a case report being a rare case. The man had full recovery after surgery and physiotherapy and was discharged home. Spontaneous bilateral quadriceps tendon rupture is a rare occurrence. The index case report is important as there was no history of trauma and it was bilateral.

## Introduction

**Figure 1:**
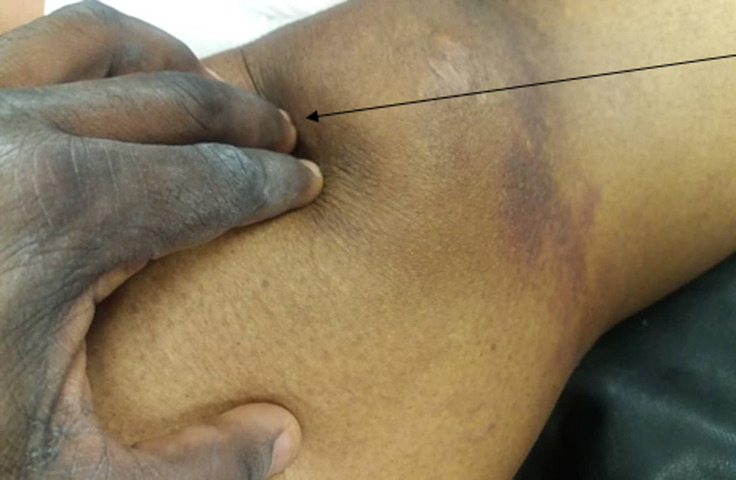
shows the dimpling at the superior pole of the patellar

**Figure 2:**
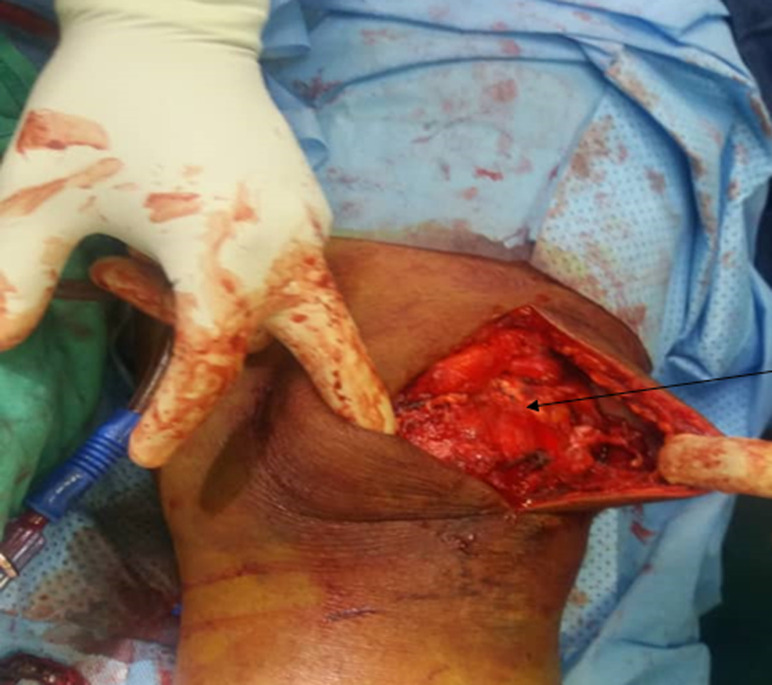
exposure of the ruptured tendon

Quadriceps tendon rupture is uncommon and occurs predominantly after major trauma among the middle-aged group involved in sport activities[1]. It can occur as a result of tendon weakening due to tendonitis, which is most common in those who participate in sports that involve jumping [1,2] Quadriceps tendon tear has been reported among patients with chronic diseases such as hyperparathyroidism [2], gout, chronic renal failure [3] and systemic lupus erythematosus [4] and those on certain long term medications such as quinolones [5], statins[1], anabolic steroids [6] and intranasal steroid [1]. It also occurs due to obesity, previous injury and fibrotic changes secondary to arteriosclerosis [1]. However, its presentation in the absence of any risk factor is yet to be reported. We report an unusual case of a 60-year old healthy man with no medical history of risk factors and on no long-term medication who presented with a spontaneous, simultaneous bilateral tendon rupture in the absence of trauma (Figure 1). Figure 2 briefly summarizes why this case is unique and may include medical literature references.

## Patient and observation

A 60 year old male businessman was standing and fell down suddenly, with difficulty innstanding, pain in both knees. No prior history of trauma. No history of steroid use or renal problems, no history of use of injections. No previous surgery.

**Clinical findings:** dimpling on the superior pole of both patellae (Figure 1), inability to straight leg raise both lower limbs, pulses intact.

**Timeline:** presented same day of incident and was placed on a backlsab splint.

**Diagnostic assessment:** diagnostic methods -MRI of the right knee performed revealed discontinuity of the distal quadriceps´ tendon 6.4mm proximal to the superior pole of the patella with mal orientation of the fibers. Retraction was also noted posteriorly. There was also partial tears of the vastus medialis and lateralis components with subcutaneous edema as well as partial tear of the patellofemoral ligament. MRI of the left knee showed partial discontinuity of the distal quadriceps´ tendon, 11mm proximal to the superior pole of the patella. Partial tears of the vastus medialis and lateralis components and the medial patellofemoral ligament was also noted. Diagnostic challenges. clinical index of suspicion. Diagnosis (including other diagnoses considered). This confirmed the diagnosis of bilateral quadriceps tendon tear as well as radiological diagnosis of partial tear of the patellofemoral ligament.

**Figure 3:**
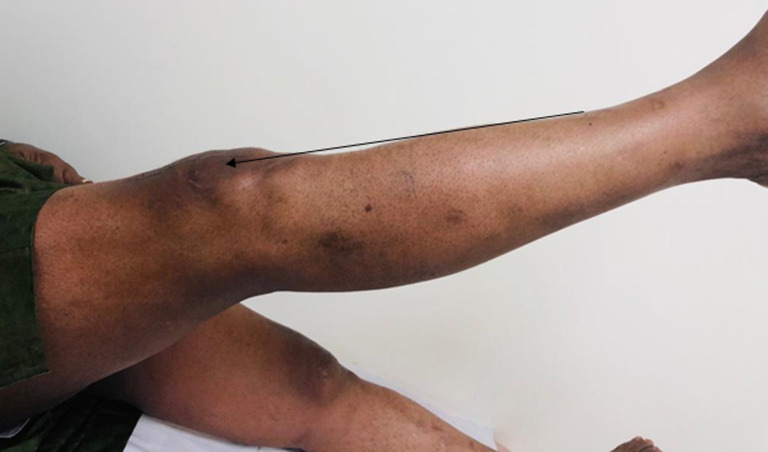
range of motion six (6) at months post-surgery

**Therapeutic intervention:** he was counselled for and subsequently underwent surgical tendon repair using Krakow method of tendon repair. Exposure of the ruptured tendon revealed full thickness rupture at the superior pole of the patella (Figure 2), Surgical repair was followed by immobilization and rehabilitation with the help of the physiotherapist. He was followed up for 6 months and he had full recovery with excellent range of motion and full straight leg raise of both lower limbs (Figure 3).

## Discussion

**Anatomy:** quadriceps femoris muscle is the largest muscle in humans and primarily functions as a knee extensor. It is made up of four muscle bellies: the rectus femoris, vastus medialis, lateralis, and intermedius [7]. These four muscles meet above the patella to form the quadriceps tendon which attaches to the superior aspect of the patella. The synchronized activity of the quadriceps femoris muscle, quadriceps tendon and patella tendon produce knee extension, therefore a complete tear disrupts this synchrony, inhibits knee extension and weight bearing of the affected limb [1,7]. Quadriceps tendon can resist considerable force. Ruptures have been reported to occur when the quadriceps are eccentrically contracted while weight bearing when the knee is in a partially flexed position [1,8]. Rupture of the quadriceps tendon is a more common knee injury after major trauma in younger patients [8]. However, rupture results from weakening of the tendon due to various factors such as obesity, arteriosclerosis-induced fibrotic changes or previous injury [1]. Quadriceps tendon rupture has been reported to occur after minor trauma in patients with chronic disease such as chronic renal failure [2], rheumatoid arthritis [9], diabetes, systemic lupus erythematosus [4], and hyperparathyroidism [2]. It has also been reported among patients on chronic use of certain medications such as steroid, quinolones [5], statin1s, anabolic steroid6 and intranasal steroid1. This is not the case with our patient. Our patient had no chronic disease, was not on any medication and he was not obese. His bilateral quadriceps tendon rupture was spontaneous with no prior or immediate trauma. His only sport activity was golf and he had not had previous surgical procedure on either knee.

**Epidemiology:** complete quadriceps tendon ruptures are rare. Incidence was reported as 1.37/100,000/year, with a mean age of 50.5 in men and 51.7 in women in UK by Clayton RA. Ninety-one percent of the quadriceps tendon ruptures occurred in males within 61-65years of age [8]. Bilateral spontaneous simultaneous rupture is rare and frequently associated with chronic conditions[10].

Diagnosis: the bilateral involvement of the tendon in the absence of trauma may lead to diagnostic confusion. Presentation of inability to walk can lead to diagnosis of proximal myopathy. The presentation of painful swelling, palpable suprapatellar gap (dimple) and the inability to actively extend the knee is found only in 60% of the cases [1,11]. However detailed history of trauma and its mechanism with evaluation of predisposing factors in conjunction with thorough examination as well as imaging can lead to accurate diagnosis. Radiological imaging used in diagnosis include plain radiograph, ultrasound and Magnetic Resonance Imaging (MRI) [1]. Plain radiograph may show intra articular swelling, shadow in the tendon line, inferior displacement of the patella and patellar spurs [1]. Suprapatellar calcification due to bony transformation can be observed in patients with systemic or degenerative diseases [1]. Ultrasound and MRI can directly prove the rupture as they visualize the soft tissue. MRI scan may also detect other intraarticular pathologies [7].

**Figure 4:**
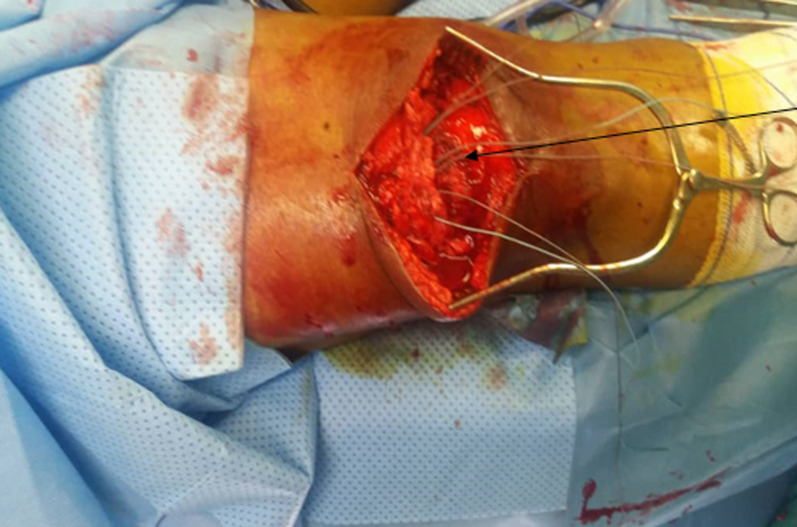
tendon repair using krakow suture method

**Treatment:** surgical repair remains the treatment of choice and involves open repair and reconstruction. Prompt repair is recommended to avoid complications such as retraction and quadriceps muscle atrophy [12]. Employed techniques in repair of tendon include end to end suture, transosseous suture and tenodesis with suture anchors. Transosseous suture is used as a gold standard repair technique, however suture anchor has been shown to provide similar clinical outcome as transosseous suture technique. Our patient underwent open repair using transosseous suture technique. This involved making 3 holes in the patella, passing Ethibond sutures through the bone tunnel and suturing the tendon to the patellar bone using the Krackow suture technique (Figure 4). Immobilization and rehabilitation post-surgical repair have shown good outcomes [12]. Post-surgery, the knees are immobilized in extension for six weeks [12]. After which gradual weight bearing and gait training is started while patient was in a knee brace [12]. The patient is weaned off knee braces with gradual increase in patient´s range of motion and knee strength. Immobilization and rehabilitation post-surgical repair have shown good outcomes with approximately two-third of patients regaining good range of motion and able to return to their previous occupation [13]. However, many patients have difficulty returning to higher-levels of sporting activities. Our patient made good recovery following surgery and rehabilitation (Figure 3). The case is important as it highlights bilateral and spontenous rupture Patient Perspective. The patient was followed up for 6 months and made significant recovery. He was happy with the outcome of treatment.

## Conclusion

Spontaneous simultaneous bilateral quadriceps tendon rupture is a rare occurrence. Our patient had no prior trauma, no chronic disease, not obese and was not on any medication shown to increases the risk of tendon rupture. We report a unique case of bilateral tendon rupture that required high index of suspicion for prompt diagnosis and management.

## References

[1] Omar M, Haas P, Ettinger M, Krettek C, Petri M (2013). Simultaneous Bilateral Quadriceps Tendon Rupture following Long-Term Low-Dose Nasal Corticosteroid Application. Case Rep Orthop.

[2] Thaunat M, Gaudin P, Naret C, Beaufils P, Thaunat O (2006). Role of secondary hyperparathyroidism in spontaneous rupture of the quadriceps tendon complicating chronic renal failure: as shown in the journal website. Rheumatology.

[3] Kim BS, Kim YW, Song EK, Seon JK, Kang KD, Kim HN (2012). Simultaneous Bilateral quadriceps Tendon Rupture in a Patient with Chronic Renal Failure. Knee Surg Relat Res.

[4] Kim BS, Kim YW, Song EK, Seon JK, Kang KD, Kim HN (2012). Simultaneous bilateral quadriceps tendon rupture in a patient with chronic renal failure: as shown in the journal website. Knee Surgery & Related Research.

[5] Calvo E, Ferrer A, Robledo AG, Alvarez L, Castillo F, Vallejo C (1997). Bilateral simultaneous spontaneous quadriceps tendons rupture. A case report studied by magnetic resonance imaging: as shown in the journal website. Clin Imaging.

[6] Potasman I, Bassan HM (1984). Multiple tendon rupture in systemic lupus erythematosus: case report and review of the literature. Ann Rheum Dis.

[7] Clayton RAE, Court-Brown CM (2008). The epidemiology of musculoskeletal tendinous and ligamentous injuries. Injury.

[8] Subhadra Nori (2018). Quadriceps tendon rupture. J Family Med Prim Care.

[9] Liow RY, Tavares S (1995). Bilateral rupture of the quadriceps tendon associated with anabolic steroids: as shown in the journal website. Br J Sports Med.

[10] Steiner CA, Palmer LH (1949). Simultaneous bilateral rupture of the quadriceps tendon: as shown in the journal website. Am J Surg.

[11] Hassani ZA, Boufettal M, Mahfoud M, Elyaacoubi M (2014). Neglected rupture of the quadriceps tendon in a patient with chronic renal failure: (case report and review of the literature). Pan Afr Med J.

[12] Senevirathna S, Radha S, Rajeev A (2011). Bilateral simultaneous rupture of the quadriceps tendon in a patient with psoriasis: a case report and review of the literature. J Med Case Rep.

[13] Plesser S, Keilani M, Vekszler G, Hasenoehrl T, Palma S, Reschl M (2018). Clinical outcomes after treatment of quadriceps tendon ruptures show equal results independent of suture anchor or transosseus repair technique used-A pilot study. PLoS One.

